# Angiosarcoma of the proximal humerus: a case report and review of the literature

**DOI:** 10.1186/1752-1947-6-347

**Published:** 2012-10-10

**Authors:** Hideki Yamashita, Koji Endo, Ryota Teshima

**Affiliations:** 1 Department of Orthopedic Surgery, Faculty of Medicine, Tottori University, 36-1 Nishi-machi, Yonago, Tottori, 683-8504, Japan

**Keywords:** Angiosarcoma, Proximal humerus, Chemotherapy, Surgery

## Abstract

**Introduction:**

Angiosarcoma of bone is an uncommon primary bone neoplasm that is composed of tumor cells that show endothelial differentiation. This is an aggressive malignancy characterized by frequent local recurrence and distant metastases. The majority of patients die within one year of diagnosis, and this shows that angiosarcoma of bone is an aggressive high-grade tumor.

**Case presentation:**

We present the case of a 65-year-old Japanese woman who had primary angiosarcoma of the proximal humerus with a pathological fracture. An open biopsy confirmed a diagnosis of primary angiosarcoma of bone. Our patient was treated with neoadjuvant chemotherapy and wide resection. One month after surgery, she developed multifocal distant metastasis to her liver and spleen.

**Conclusions:**

Angiosarcoma of the humerus is extremely rare. Radiographically, there is no specific finding associated with angiosarcoma of bone as opposed to other malignant bone tumors. The cornerstone of treatment is en bloc resection followed by as much adjuvant radiation therapy as possible. However, the role of chemotherapy remains undefined, and better systemic agents are clearly needed.

## Introduction

Malignant vascular tumors of bone are very rare and account for less than 1% of primary malignant bone tumors. Angiosarcoma is an uncommon neoplasm characterized by rapidly proliferating and extensively infiltrating anaplastic cells derived from vessels and lining irregular blood-filled spaces [1]. Skeletal angiosarcoma generally affects young adults and elderly individuals, and age distribution shows a wide range with a nearly equal distribution from the second to the eighth decade of life [2]. Angiosarcomas tend to affect long tubular bones of the extremities and the axial skeleton, mainly the spine. The bones of the lower limb, particularly the femur and the tibia, are most commonly involved, followed by the pelvis, vertebral column, and the bones of the upper limbs [3,4]. We describe an extremely rare case of a woman with angiosarcoma of the humerus who underwent chemotherapy and wide resection.

## Case presentation

**Figure 1:**
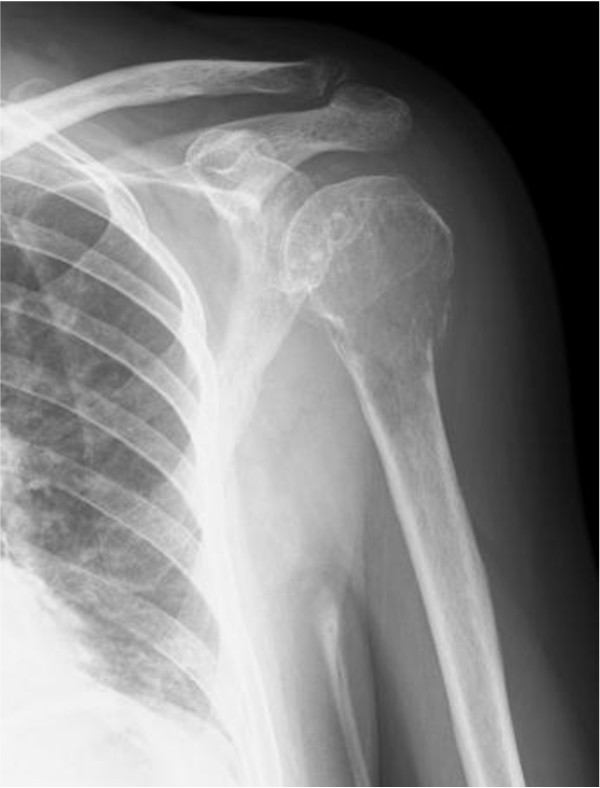
Anteroposterior radiograph of the left shoulder shows a pathological fracture with an osteolytic lesion.

**Figure 2:**
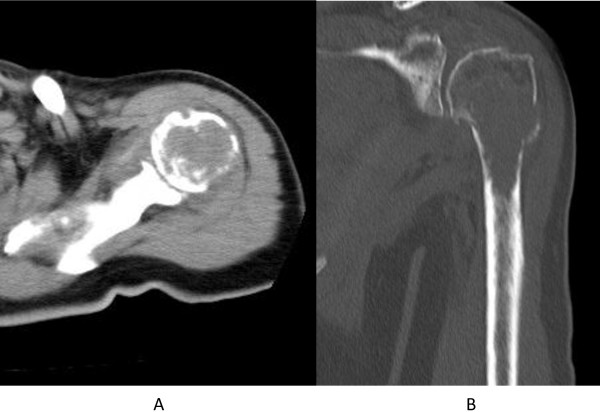
**Computed tomography scan shows cortical destruction of the proximal humerus and a pathological fracture.** (**A**) Transverse view. (**B**) Coronal view.

**Figure 3:**
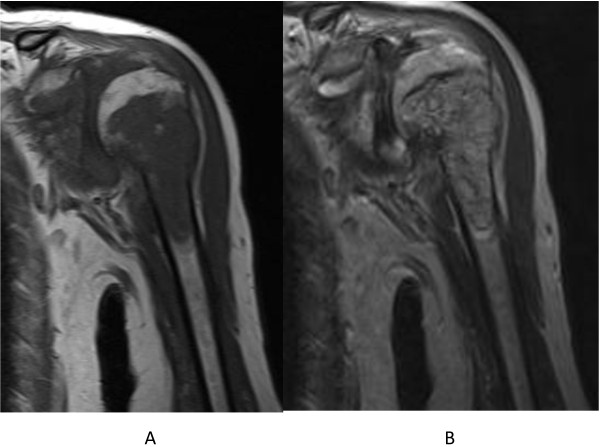
**Magnetic resonance imaging (MRI) of the left proximal humerus.** (**A**) Sagittal T1-weighted MRI shows a low-signal-intensity tumor replacing bone marrow. (**B**) Coronal T2-weighted MRI demonstrates an increase in the signal intensity of the tumor.

**Figure 4:**
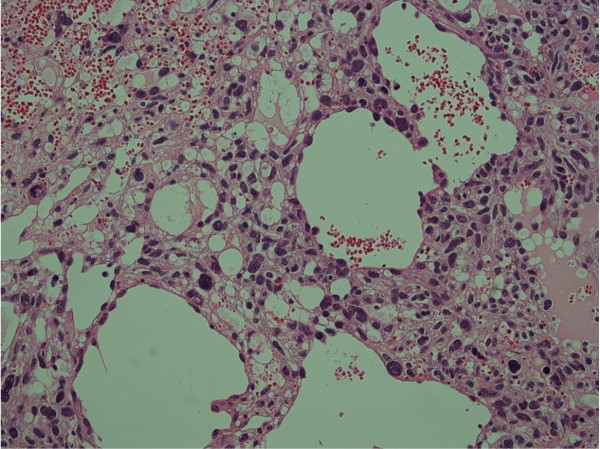
**A tumor composed of anastomosing vascular channels and cystic spaces lined by malignant cells is seen.** Stain: hematoxylin and eosin; original magnification: ×200.

**Figure 5:**
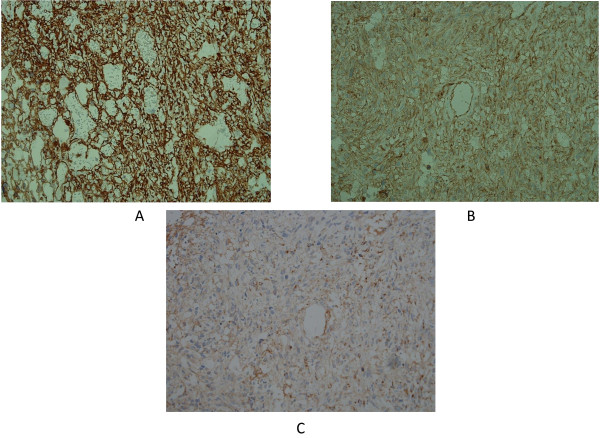
**Immunohistochemical analysis revealed strong staining of tumor cells for CD31, CD34, and factor VIII RA.** (**A**) CD34. (**B**) CD31. (**C**) Factor VIII RA. Original magnification: × 100.

A 65-year-old Japanese woman fell as she was getting out of bed and injured her left shoulder. Plain radiograph and computed tomography (CT) revealed an osteolytic lesion in her proximal humerus with a wide zone of transition between the tumor and uninvolved bone (Figures 1 and 2). The cortex of her proximal humerus was thinned, and a pathological fracture was also seen, but cortical expansion was absent in that lesion. Magnetic resonance imaging showed a lesion with low intensity on T1-weighted images and intermediate intensity on T2-weighted images (Figure 3). Gadolinium-enhanced T1-weighted images showed heterogeneous enhancement of the lesion. Fluorine-18-fluorodeoxyglucose positron emission tomography imaging demonstrated no distant metastases. Open biopsy revealed a tumor composed of anastomosing vascular channels and cystic spaces lined by malignant cells (Figure 4). Immunohistochemical analysis showed CD31, CD34, and factor VIII RA positivity, confirming the vascular origin of the tumor, and a diagnosis of primary angiosarcoma of bone was made (Figure 5).

Our patient was treated with three cycles of pre-operative chemotherapy composed of mesna, doxorubicin, ifosfamide, and dacarbazine, which is the MAID protocol. During the three cycles of pre-operative chemotherapy, the size of the mass did not change, but her spontaneous pain decreased.

**Figure 6:**
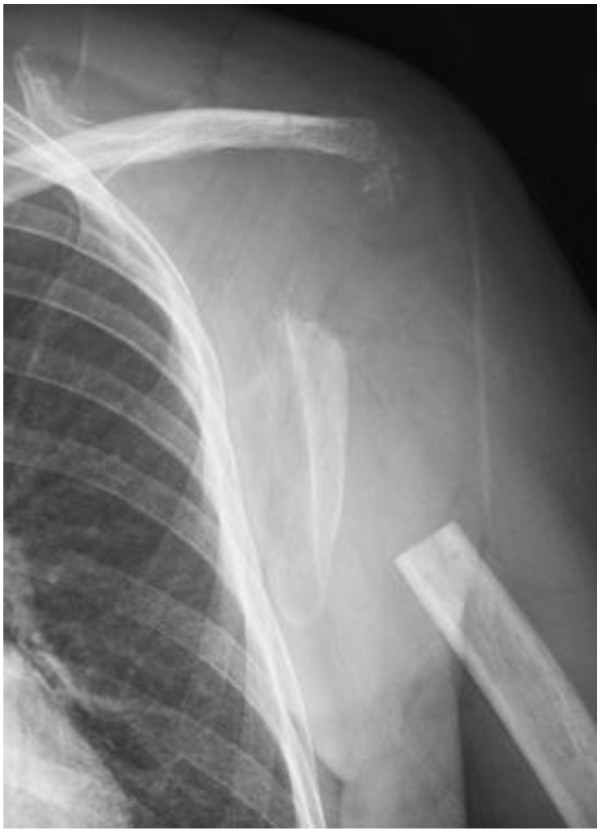
Immediate post-operative radiograph of the left shoulder after wide resection.

**Figure 7:**
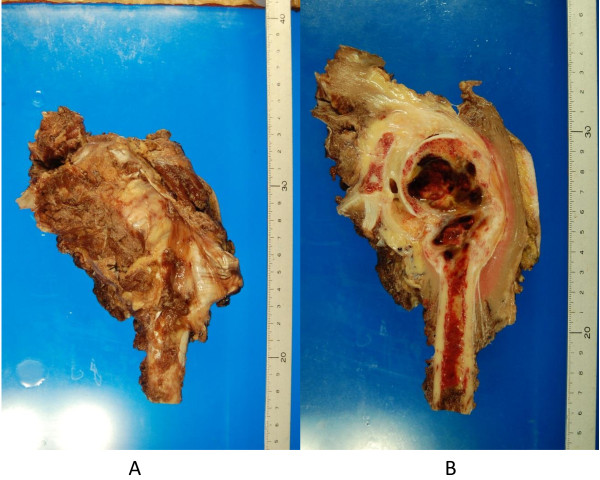
**Excised specimen of the proximal humerus.** (**A**) Macroscopic appearance of resected specimen. (**B**) Coronal cut surface of the specimen.

After pre-operative chemotherapy, en bloc resection of the tumor was performed by extra-articular humeral and glenoid resection (Figures 6 and 7). The clavicula pro humero method was initially planned for reconstruction, but the acromioclavicular joint was collapsed, and the distal clavicular end detached from surrounding soft tissue during osteotomy of the lower aspect of acromion. Therefore, reconstruction with this method failed, resulting in a flail shoulder. After surgery, our patient did not wish to receive additional reconstruction surgery and adjuvant radiotherapy. Two months after surgery, she had moderate function according to a Musculoskeletal Tumor Society and International Symposium on Limb Salvage [5] evaluation; she had scores of five for pain, one for function, three for emotional acceptance, one for positioning of the hand, five for manual dexterity, and zero for lifting ability. The total score was 15 points (50%). Our patient did not complain of any pain. Impairment of activity of daily living was slight when a sling was used.

**Figure 8:**
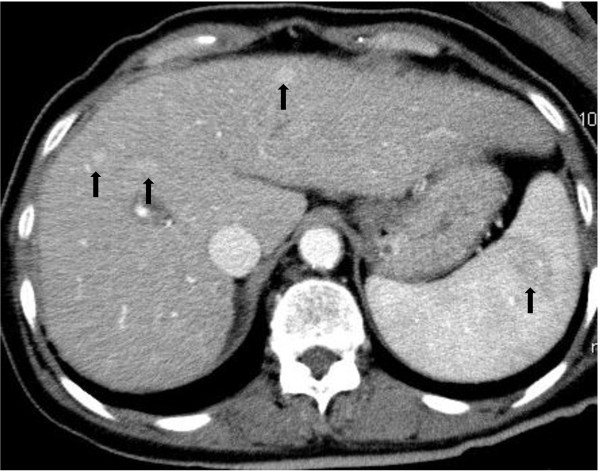
Contrast-enhanced computed tomography scan shows metastatic tumors in the liver and spleen (arrows).

Histological examination of the excised tumor indicated that the surgical margin was wide in all directions, and necrosis was observed in more than approximately 90% of the maximum cut surface. After surgery, our patient's upper limb was immobilized with a stockinette-Gilchrist bandage. Four weeks after surgery, CT scans of her abdomen revealed distant metastases in her liver and spleen (Figure 8). One cycle of post-operative chemotherapy using the MAID protocol was performed, but she discontinued additional post-operative chemotherapy. We did not see local recurrence until the final follow-up. Five months after surgery, she transferred to another hospital because she had changed residence.

## Discussion

Angiosarcoma of bone is a rare malignant neoplasm and represents the most malignant end of the spectrum of vascular tumors. Approximately 6% of all angiosarcomas are found in bone [2]. To the best of our knowledge, angiosarcoma of the humerus is extremely rare; only five cases have been reported in the literature in English since 1953 [6-10] (Table 1). This is an aggressive malignancy characterized by frequent local recurrence and distant metastases. These lesions occur during the second to the seventh decades of life, although the peak is during the third to fifth decades [11]. Men are affected with twice the frequency of women. The most common osseous lesions are the long bones (approximately 60% of cases), particularly the tibia (23%), femur (18%), and humerus (13%), as well as the pelvis (7%) [12]. The two most common symptoms are local pain and swelling.

**Table 1:** Reported cases of angiosarcoma of the humerus

**Case number**	**Reference**	**Patient**	**Radiographic features**	**Treatment**	**Outcome**
1	Hasegawa *et al*. [8]	48-year-old man	A multilocular osteolytic lesion with undefined margins and destroyed cortical and medullary bone	Amputation of the arm at the level of the scapula	DOD
2	Fukuroku *et al*. [7]	55-year-old man	Severe bone destruction of diaphysis of humerus by tumor invasion	Interscapulothoracic amputation	DOD
3	Voggenreiter *et al*. [10]	12-year-old boy	ND	Wide resection	CDF
4	Chen [6]	87-year-old man	Expansile lytic lesion involving the entire diaphysis of the humerus with a fracture in the distal portion of the lesion	Disarticulation of the shoulder	ND
5	Mittal *et al*. [9]	67-year-old man	Erosion of medial cortex with lytic areas at upper end of humerus	Wide resection and adjuvant chemotherapy	CDF

CDF: continuously disease-free; DOD: dead of disease; ND: not documented.

Radiographically, there is no specific finding associated with angiosarcoma of bone as opposed to other malignant bone tumors. Lesions are usually highly destructive and may grow too fast to invoke a periosteal reaction. They may be eccentric and may have a purely lytic or mixed lytic sclerotic pattern [13]. More aggressive features include osseous expansion, cortical permeation, and an associated soft tissue mass [14]. CT and magnetic resonance imaging characteristics of angiosarcoma may also be non-specific [14]. In general, pathological fractures of high-grade primary bone sarcomas occur either spontaneously or after minimal trauma. The incidence of pathological fracture at diagnosis in patients with angiosarcoma of bone is unknown.

Pathologically, these tumors may contain either hemangiomatous or lymphangiomatous cellular elements, which are often difficult or impossible to distinguish histologically, particularly with higher degrees of anaplasia. Identification of vascular channels allows diagnosis; thus, the term angiosarcoma is preferable to hemangiosarcoma or lymphangiosarcoma for these malignant vascular neoplasms [15]. Verbeke *et al*. [16] recently reported that primary angiosarcoma of bone exhibiting more than three mitoses per 10 high-powered fields (HPFs), a prominent nucleolus, and fewer than five eosinophilic granulocytes per 10 HPFs has a more aggressive course and worse outcome, indicating that these histological criteria have prognostic value.

In angiosarcoma, local recurrence is frequent, prognosis is notoriously poor, and early metastases develop. Metastases to the lungs and other parenchymal organs are found in about 66% of cases [15]. It is reported that the majority of patients die within one year of diagnosis, and this shows that angiosarcoma of bone is an aggressive high-grade tumor [17]. In our case, one month after surgery, multiple distant metastases were observed, despite three cycles of pre-operative chemotherapy (MAID).

The treatment of angiosarcoma is often quite challenging. The cornerstone of treatment is en bloc resection followed by as much adjuvant radiation therapy as possible [18]. Surgery combined with immediate post-operative radiation therapy could be an optimal treatment method, even if complete resection is possible [19]. In our patient, adjuvant radiotherapy was not performed, because she refused.

The role of chemotherapy remains undefined [19]. Several anticancer drug combinations, including cisplatin with doxorubicin, cisplatin plus paclitaxel, and cisplatin plus doxorubicin plus paclitaxel, have been investigated [20]. Abraham *et al*. [13] reported that doxorubicin-based chemotherapy regimens, which are commonly used for other sarcoma subtypes, have also been used for angiosarcomas. Patients were generally treated with doxorubicin-based regimens or vinorelbine, and 64% of patients had at least a partial response to at least one regimen. Given these data, a combination of mesna, doxorubicin, ifosfamide, and dacarbazine – that is, the MAID protocol – was administered to our patient before and after surgery. However, post-operative CT scans revealed distant metastases in her liver and spleen. Prolonged responses were uncommon, and better systemic agents are clearly needed.

Recently, a novel t (1; 14) (p21; q24) translocation was described in angiosarcoma of bone [21]. To the best of our knowledge, this is the first cytogenic aberration reported in angiosarcoma of bone. However, small series have shown the involvement of tumor-suppressor genes such as *p53* and *p16*, mainly in angiosarcoma of soft tissue, suggesting a possible role in tumorigenesis in a subset of angiosarcomas. It is still unclear whether primary angiosarcoma of bone is a true separate entity or is similar to primary angiosarcoma of deep soft tissues. Further molecular studies might be required in order to investigate the different therapeutic targets.

## Conclusions

We present an extremely rare case of angiosarcoma of bone with a pathological fracture. Our case adds to the limited literature on angiosarcoma of the humerus. The cornerstone of treatment is en bloc resection followed by as much adjuvant radiation therapy as possible. However, the role of chemotherapy remains undefined, and better systemic agents are clearly needed.

## Consent

Written informed consent was obtained from the patient for publication of this case report and the accompanying images. A copy of the written consent is available for review by the Editor-in-Chief of this journal.

## Abbreviations

CT: Computed tomography; HPF: High-powered field; MAID: Mesna, doxorubicin, ifosfamide, and dacarbazine.

## Competing interests

The authors declare that they have no competing interests.

## Authors' contributions

HY helped to prepare the case report, perform a literature search on angiosarcoma of bone, and write the case report and discussion. KE helped to prepare the case report and perform a literature search on angiosarcoma of bone. RT helped to write the case report and discussion. All authors read and approved the final manuscript.
